# Titania/Electro-Reduced Graphene Oxide Nanohybrid as an Efficient Electrochemical Sensor for the Determination of Allura Red

**DOI:** 10.3390/nano10020307

**Published:** 2020-02-11

**Authors:** Guangli Li, Jingtao Wu, Hongguang Jin, Yonghui Xia, Jun Liu, Quanguo He, Dongchu Chen

**Affiliations:** 1 College of Life Sciences and Chemistry, Hunan University of Technology, Zhuzhou 412007, China; Jtwu123@163.com (J.W.); junliu@hut.edu.cn (J.L.); hequanguo@126.com (Q.H.); 2 School of Materials Science and Energy Engineering, Foshan University, Foshan 528000, China; 3 College of Materials Science and Engineering, Changsha University of Science and Technology, Changsha 410114, China; jesonjin08@csust.edu.cn; 4 Zhuzhou Institute for Food and Drug Control, Zhuzhou 412000, China; sunnyxia0710@163.com

**Keywords:** TiO_2_, electro-reduced graphene oxide, Allura Red, voltammetric sensor

## Abstract

Titania/electro-reduced graphene oxide nanohybrids (TiO_2_/ErGO) were synthesized by the hydrolysis of titanium sulfate in graphene oxide suspension and in situ electrochemical reduction. It provides a facile and efficient method to obtain nanohybrids with TiO_2_ nanoparticles (TiO_2_ NPs) uniformly coated by graphene nanoflakes. TiO_2_/ErGO nanohybrids were characterized by transmission electron microscopy, X-ray diffraction, cyclic voltammogram, and electrochemical impedance spectroscopy in detail. Compared with pure ErGO and TiO_2_ NPs, TiO_2_/ErGO nanohybrids greatly enhanced the electrocatalytic activity and voltammetric response of Allura Red. In the concentration range of 0.5–5.0 μM, the anodic peak currents of Allura Red were linearly correlated to their concentrations. However, the linear relationship was changed to the semi-logarithmic relationship at a higher concentration region (5.0–800 μM). The detection limit (LOD) was 0.05 μM at a signal-to-noise ratio of 3. The superior sensing performances of the proposed sensor can be ascribed to the synergistic effect between TiO_2_ NPs and ErGO, which provides a favorable microenvironment for the electrochemical oxidation of Allura Red. The proposed TiO_2_/ErGO/GCE showed good reproducibility and stability both in determination and in storage, and it can accurately detect the concentration of Allura Red in milk drinks, providing an efficient platform for the sensitive determination of Allura Red with high reliability, simplicity, and rapidness.

## 1. Introduction

Nowadays, the safety of food additives has become an increasingly important concern worldwide, since most of these substances are poisonous and even carcinogenic. Among diverse food additives, synthetic colorants have been extensively added into various soft beverages and foodstuffs to make them more attractive and appealing. However, some synthetic colorants can lead to adverse health issues, especially if they are excessively consumed [1,2]. Therefore, the use of synthetic colorants in foodstuffs were rigorously controlled by legislation all over the world. Allura Red (E 129) is a typical water-soluble azo dye, which was usually added to candies, syrups, and beverages to make a fascinating red color. Despite its widespread applications, the health threats cannot be underestimated, since its aromatic rings and azo groups may harm the human body. The European Food Safety Authority (EFSA, Parma, Italy) recommended that the acceptable daily intake (ADI) of Allura Red is 7.0 mg/kg bw/day [3]. In China, the maximum permitted dosage in soft beverages is 0.1 g/kg referring to the national standard GB 2760-2014 [4]. Unfortunately, the dosages are frequently exceeded [5]. In Chinese regulation, the added colorants must be listed on commercial food labels, but their concentrations are not required. Therefore, it attaches great importance to detect Allura Red in foodstuffs to know their consumption, which is mainly due to the large and prolonged consumption of colorful foodstuffs and beverages. 

Up to now, several analytical methods have been established for the routine detection of Allura Red, including spectrophotometry [2,6], high-performance liquid chromatography (HPLC) [7,8,9], cloud point extraction [10,11], and capillary electrophoresis [12,13]. However, these analytical techniques are not always available and involve complicated and time-consuming pretreatment procedures that require expensive instruments and toxic solvents. In recent years, electroanalytical techniques have attracted growing attention and showed promising applications in sensing bioactive molecules and food contaminants due to their portability, cost-effective, rapid response, facile operation, and high sensitivity [14,15,16,17,18,19,20,21]. Therefore, it is feasible to miniaturize the devices for in situ determination. Allura Red contains an –N=N– group, which is electrochemically active and can be reduced at the mercury electrodes [22,23]. Although these mercury electrodes have low detection limits, toxic mercury electrodes are not environmentally friendly and cause adverse health problems. Moreover, their reduction signals are susceptible to the soluble oxygen, which must be completely removed from the solution before electrochemical determination. Recently, some environmentally friendly mercury-free electrodes such as glassy carbon [24], antimony, and bismuth film electrodes [25,26,27] have been developed for the electrochemical detection of Allura Red. However, these mercury-free electrodes showed worse sensitivity, and cathodic peaks occurred at more negative potential compared to mercury electrodes. To address the above-mentioned issues, much effort has been made to develop novel modified electrodes that are capable of efficiently detecting Allura Red based on the oxidation signals. Novel electrochemical sensors based on multiwalled carbon nanotube (MWCNT) [28,29], ionic liquids [30], cobalt oxide [31], and cobalt complex [32] modified electrodes have shown great potentials on the detection of Allura Red, due to their excellent accumulation efficiency and large specific surface area. For example, MWCNT/GCE showed a wide linear response range of 496–4468 μg/L, and it was applied to the analysis of commercial isotonic sports drinks with satisfactory results [28]. The cobalt complex-decorated carbon paste electrode exhibited a wide linear response range of 60–1489 μg/L, and it was successfully applied in foods such as orange and strawberry-flavored gelatin, powders, soft drinks, and pharmaceutical dosages [32]. However, the anodic peaks generally appeared at a rather positive potential (above 0.7 V), which may cause significant interfering from some species that are easily oxidized at such a high potential. In addition, some of the modification materials are not readily available and very expensive. Hence, finding more available, low-cost sensing materials that are capable of the electrocatalytic oxidation of Allura Red at lower potential remains challenging. 

Titania nanoparticles (TiO_2_ NPs) have become versatile materials for various (photo) electrochemical sensors and photocatalysts, owing to their fascinating properties including greater availability, low cost, good conductivity, strong adsorptive capacity, and efficient catalytic properties [33,34,35,36,37,38,39,40,41,42]. TiO_2_ NPs can be easily anchored on the surface of metal electrodes [43], but immobilizing them directly on the surface of glass carbon electrodes (GCE) is difficult. Due to their unique properties such as their large surface to-volume ratio, extraordinary electrical conductivity, and rapid electron transfer rate [44,45,46,47,48], graphene (Gr) has been considered as the most preferred electrode material for electrochemical sensors. Its unique 2D crystal structure and electronic property endows itself an ideal catalyst support for the anchoring of metal or metal oxide NPs [49], which offers versatile selective catalytic or sensing properties. Moreover, Gr can be easily decorated on the surface of GCE via π–π interaction. Hence, graphene is a suitable support to disperse and stabilize TiO_2_ NPs. For these reasons, TiO_2_/Gr nanohybrids have exhibited greater versatility as advanced sensing materials for the sensitive detection of adenine [36], guanine [36], glucose [50], dopamine [51], paracetamol [52], L-tryptophan [53], L-tyrosine [53], etc. However, to our best knowledge, TiO_2_/Gr nanohybrids have been rarely used for the detection of Allura Red.

Herein, TiO_2_/Gr nanohybrids were synthesized for the sensitive detection of Allura Red in soft drinks. TiO_2_/electro-reduced graphene oxide nanohybrids (TiO_2_/ErGO) were synthesized by the hydrolysis of titanium sulfate in graphene oxide (GO) suspension and in situ electrochemical reduction. In this way, the oxygen-containing functional groups present in graphene oxide can be reduced with the application of the appropriate potential. The electrical conductivity was greatly improved due to the restoration of carbon-conjugated networks after electrochemical reduction. Compared with chemical and thermal reduction methods, the electrochemical reduction is very simple and time-saving. Moreover, no any poisonous and potentially explosive chemicals such as hydrazine and sodium borohydride are not required. The synergistic effect between TiO_2_ NPs and ErGO nanosheets greatly improved the electrochemical oxidation of Allura Red, with remarkably enhanced voltammetric response and decreased anodic peak potential. Moreover, the proposed TiO_2_/ErGO exhibited good selectivity, sensitivity, and stability for the detection of Allura Red.

## 2. Materials and Methods 

### 2.1. Chemicals

Allura Red, amaranth, K_3_[Fe(CN)_6_], K_4_[Fe(CN)_6_], KCl, Na_2_HPO_4_, and NaH_2_PO_4_ were supplied by Aladdin Reagents Co., Ltd (www.aladdin-e.com, Shanghai, China). All the reagents were of analytical grade and directly used as received. GO was purchased from Xianfeng NANO Material Tech Inc. (Nanjing, China). Liziyuan milk drinking was purchased from a local supermarket. 

### 2.2. Prepration of TiO_2_/GO Nanocomposites

TiO_2_ NPs were synthesized by the hydrolysis of titanium sulfate in GO suspension. Typically, 40 mg of GO was dispersed in a mixed solvent of ethanol (10 mL) and water (20 mL) under 0.5 h of ultrasonication to obtain a homogenous suspension of exfoliated GO. Then, 6.2 mg of titanium sulfate was added to the GO suspension and ultrasonicated for 0.5 h. Afterwards, the resultant solution was transferred to a 50-mL autoclave and reacted at 180 °C for 4 h. After it was cooled to room temperature, the reaction solution was centrifuged at a rate of 11,000 r/min for 10 min. Subsequently, the solids were repeatedly washed with absolute ethanol and deionized water for 2–3 rounds; then, they were vacuum-dried at 60 °C overnight. The TiO_2_/GO nanohybrids were obtained as black powder. The as-obtained TiO_2_/GO nanohybrids (10 mg) were ultrasonically dispersed in 10 mL of deionized water for 2 h to get a homogenous dispersion (1 mg/mL).

### 2.3. Preparation of TiO_2_/ErGO Modified Electrodes

Prior to electrode modification, bare GCEs were carefully polished to mirror-like surface at a cloth pad using 0.05 μm Al_2_O_3_ slurry; then, they were alternately cleaned by anhydrous alcohol and deionized water. The freshly polished GCEs were dried under an infrared lamp. TiO_2_/ErGO/GCE were prepared by a simple drop-casting and in situ electrochemical reduction technique. Typically, 5 μL of TiO_2_/GO dispersion was dropped and casted on the surface of GCEs; then, it was allowed to dry under an infrared lamp. Finally, the TiO_2_/GO/GCEs were immersed into 0.1 M PBS solution (pH = 6.0) and electrochemically reduced at −1.2 V for 2 min. For comparison, individual TiO_2_ NPs and ErGO-modified GCEs were also manufactured by the same procedure.

### 2.4. Electrochemical Detection of Allura Red

A classical three-electrode assembly was used in all the electrochemical measurements, consisting of a bare or modified GCE, a saturated calomel electrode (SCE), and a platinum wire as the working electrode, reference electrode, and auxiliary electrode, respectively. First, 0.1 M of phosphate buffer solution (PBS, pH 7.0) was used as the supporting electrolyte unless otherwise stated. To assess the electrochemical properties of the proposed sensor, cyclic voltammogram and electrochemical impedance spectroscopy was recorded in 0.1 mM [Fe(CN)_6_]^3−/4−^ probe solution. Electrochemical impedance spectroscopy was scanned from 100,000 to 0.1 Hz at open circuit potential using a 5 mV (rms) AC sinusoid signal. After a suitable accumulation, the differential pulse voltammetry (DPV) was measured from 0.3 to 0.9 V. The pulse amplitude is 0.05 V, the pulse width is 0.05 s, the sample width is 0.0167 s, and the pulse period is 0.2 s. 

## 3. Results and Discussions

### 3.1. Materials Chararazation

**Figure 1:**
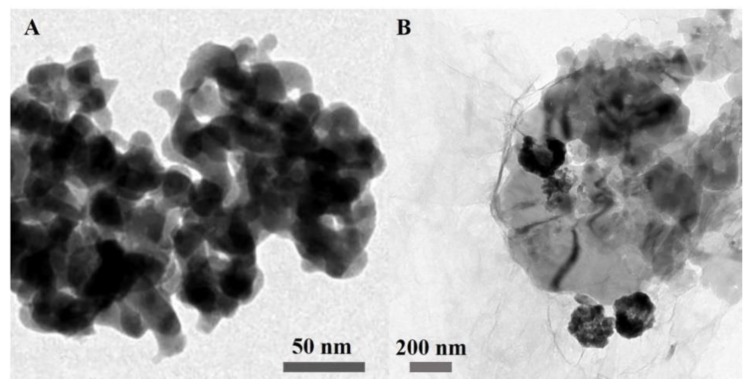
TEM images of TiO_2_ nanoparticles (TiO_2_ NPs) (**A**) and electro-reduced graphene oxide nanohybrids (TiO_2_/ErGO) nanohybrids (**B**).

The microstructures of TiO_2_ NPs and TiO_2_/ErGO nanohybrids were investigated by transmission electron microscopy (TEM). Cube-like structures were observed in Figure 1A, and the particle sizes of TiO_2_ NPs are estimated to 30–50 nm. After introducing ErGO sheets onto the surface of TiO_2_ NPs, TiO_2_ NPs were wrapped and coated by ErGO nanoflakes (Figure 1B), confirming that TiO_2_ NPs were successfully hybridized with ErGO nanoflakes. 

**Figure 2:**
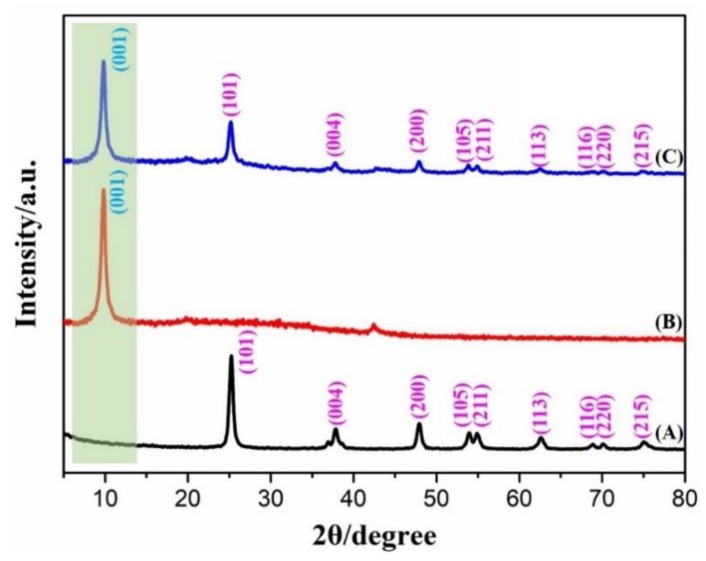
XRD patterns of TiO_2_ NPs (**A**), graphene oxide (GO) nanosheets (**B**), and TiO_2_/GO nanohybrids (**C**).

The crystalline structures of TiO_2_ NPs, GO nanoflakes, and TiO_2_/GO nanohybrids were further investigated by X-ray diffraction (XRD). As shown in Figure 2A, pronounced diffraction peaks appear at 25.26°, 36.92°, 47.94°, 53.98°, 54.90°, 62.60°, 68.86°, 70.08°, and 75.06°, respectively, which can be well assigned to (101), (004), (200), (105), (211), (213), (116), (220), and (215) crystal planes of anatase TiO_2_ (JCPDS card 21-1272), respectively. Moreover, no visible impurity peaks were observed. The results suggest that the as-obtained TiO_2_ NPs have anatase structure with good crystallinity. The XRD pattern of GO shows a sharp diffraction peak at 2Ɵ = 10.6° (Figure 2B), which can be indexed to the (001) crystal plane of GO with a d-spacing of 0.80 nm [54]. In the XRD pattern of the TiO_2_/GO nanocomposite in Figure 2C, the major characteristic peaks of GO can also be identified in addition to TiO_2_ peaks. The results indicate that the TiO_2_/GO nanocomposite was successfully prepared.

**Figure 3:**
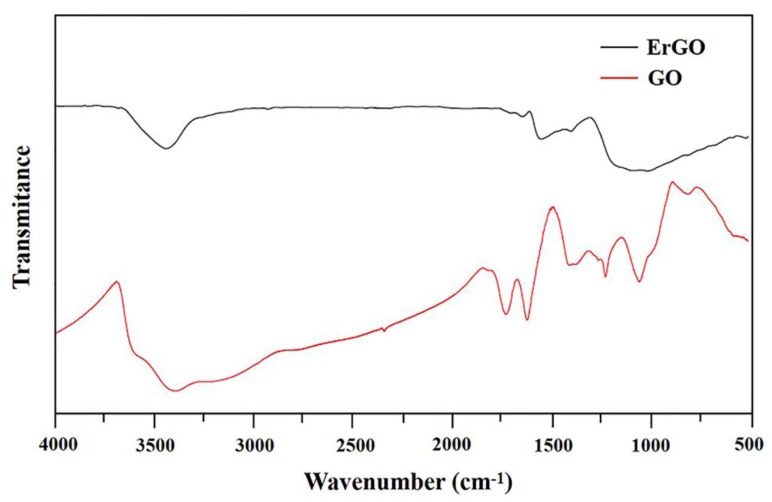
FTIR spectra of graphene oxide (GO) and electro-reduced graphene oxide (ErGO).

The Fourier transform infrared spectroscopy (FTIR) of GO and ErGO are shown in Figure 3. In the FTIR of GO, apparent peaks at 1725, 1618, 1407, 1225, and 1057 cm^−1^ are assigned to the characteristic peaks of C=O (carbonyl or carboxy), C=C (aromatics), C-O (carboxy), C-O (epoxy), and C-O (alkoxy), respectively. As expected, these oxygen-containing functional groups almost disappear, indicating that graphene oxide was successfully electrochemically reduced into reduced graphene oxide.

### 3.2. Assessement of Electrochemical Properties

**Figure 4:**
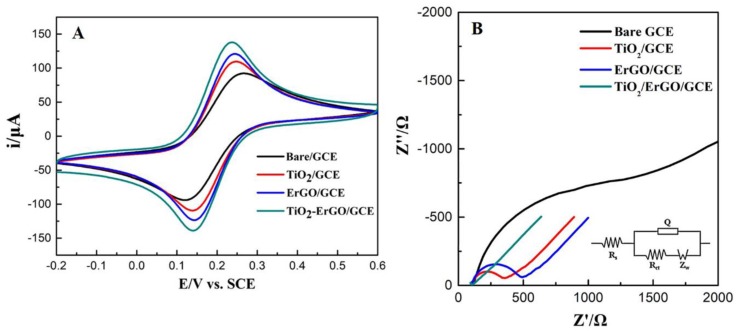
Cyclic voltammograms (**A**) and Nyquist plots (**B**) measured at different electrodes in 0.1 mM [Fe(CN)_6_]^3−/4−^ and KCl mixture solution. The inset in Figure 4B represents the equivalent circuit model.

To assess the electrochemical properties, cyclic voltammograms for different modified electrodes were recorded in 0.1 M [Fe(CN)_6_]^3−/4−^ and KCl mixture solution. As shown in Figure 4A, the ratios of anodic and cathodic peak currents (i_pa_/i_pc_) approach to 1 for all electrodes, suggesting that all electrodes experience a quasi-reversible process. The redox peak currents increase in the following order: bare GCE, TiO_2_/GCE, ErGO/GCE, and TiO_2_/ErGO/GCE. Among these electrodes, TiO_2_/ErGO/GCE shows the largest electrochemical response, with i_pa_ and i_pc_ of 148.2 and 144.1 μA, respectively. Moreover, the peak separation between anodic and cathodic peaks (∆E_p_ = 67 mV) is the smallest. All these results demonstrate the synergistic effect between TiO_2_ NPs and ErGO. Referring to the Randles–Sevcik equation [18,46], the effective electroactive areas of bare GCE, TiO_2_/GCE, ErGO/GCE, and TiO_2_/ErGO/GCE were estimated to 0.0768, 0.0959, 0.1052, and 0.1308 cm^2^, respectively. The effective electroactive area of TiO_2_/ErGO/GCE is twice of that of bare GCE approximately. The results indicate that TiO_2_/ErGO nanohybrids greatly increase the electroactive surface area, which will eventually boost the electrochemical responses.

Electrochemical impedance spectroscopy is a valuable tool to investigate the charge transfer ability between electrode and electrolyte interfaces [18,46,55,56]. Nyquist plots includes the semicircular at high frequencies and linear portions at low frequencies, which are closely associated with the electron transfer-limited process and diffusion-controlled process, respectively. Generally, the charge transfer resistance (R_ct_) equals a semicircle diameter. As shown in Figure 4B, the R_ct_ value decreases from 1562 Ω to 341 Ω and 211 Ω after loading ErGO nanosheets and TiO_2_ NPs onto the bare electrode, respectively, which means that the charge transfer between the electrode and solution is more efficient after the modification with electrode by ErGO and TiO_2_ NPs. In other words, both TiO_2_ NPs and ErGO nanosheets can significantly improve the electron transfer efficiency. As expected, a notable decrease in the semicircle diameter (R_ct_ = 14.5 Ω) is found on the TiO_2_/ErGO/GCE. It is believed that the remarkably decreased R_ct_ can promote the electrochemical sensing performance.

### 3.3. Electrochemical Response of Allura Red on Different Modified Electrodes

**Figure 5:**
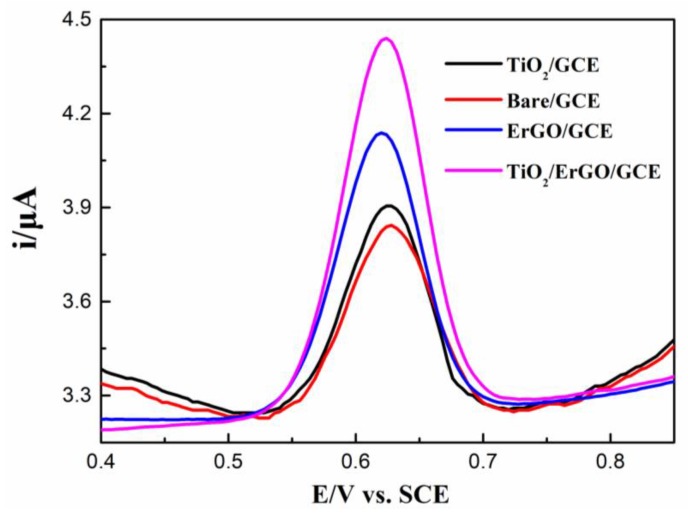
Differential pulse voltammetry (DPVs) of 100 μM Allura Red recorded at different modified electrodes.

DPV responses of 100 μM Allura Red were recorded at different electrodes (Figure 5). Clearly, the anodic peak current of Allura Red at bare GCE is weakest (i_pa_ = 0.607 μA). After introducing TiO_2_ NPs, the anodic peak current of Allura Red increased to 0.768 μA due to the good electrocatalytic activity and high adsorptive ability [36,50,51,52]. When the GCE was decorated with TiO_2_/ErGO nanohybrids, the anodic peak current of Allura Red greatly increases (i_pa_ = 0.890 μA), owing to the combined effect from good electrical conductivity and high specific surface area, which facilitates the absorption efficiency and electrochemical reactivity of Allura Red [52]. As expected, TiO_2_/ErGO/GCE shows the largest response signal (i_pa_ = 1.187 μA). The anodic peak potentials (E_pa_) of bare GCE, TiO_2_/GCE, ErGO/GCE, and TiO_2_/ErGO/GCE are 0.648, 0.642, 0.624, and 0.620 V, respectively. Interestingly, the anodic peak potential at TiO_2_/ErGO/GCE is lower compared with previous reports [28,29,32,57], which can effectively avoid the co-oxidation of other interferences that may coexist in food with Allura Red. It probably because TiO_2_/ErGO nanohybrids can effectively accelerate the electron transfer between and analyte, thus minimizing the activated polarization potential. The enhancement on the peak current and lowering of oxidation overpotential demonstrate that TiO_2_/ErGO nanohybrids have superior electrocatalytic activity toward the oxidation of Allura Red. It can be reasonably ascribed to the synergistic effect between TiO_2_ NPs and ErGO nanosheets, which combines the unique properties of two individual components. The electrocatalytic effect and cumulate accumulation capability of TiO_2_/ErGO nanohybrids accelerate electron transfer and eventually improve the electrochemical sensing performances [36,50,51,52,53]. 

### 3.4. Optimization of Analytical Conditions

#### 3.4.1. Effect of pH

**Figure 6:**
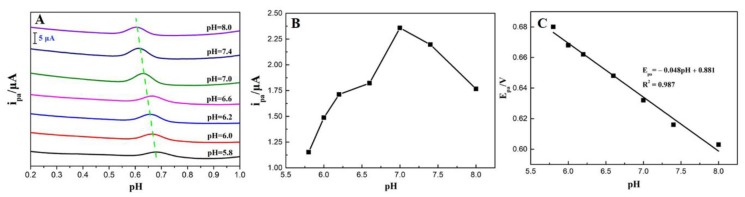
(**A**) DPV curves of Allura Red recorded at various pH; (**B**) The influence of pH on the anodic peak current of Allura Red; (**C**) Linear relationship between anodic peak potential and pH.

The effect of pH on the response anodic peak current of Allura Red was explored by DPV. As displayed in Figure 6A, the anodic peaks of Allura Red shifts toward a negative potential direction, which demonstrate the proton participant in the redox process of Allura Red. The anodic peak currents of Allura Red gradually increase with pH varying from 5.6 to 7.0; then, they slowly decrease when the pH exceeds 7.0 (Figure 6B). Therefore, pH 7.0 was selected as the optimal pH. In addition, the anodic peak potentials of Allura is inversely related to pH, with the linear expression of *E*_pa_ = −0.048 pH + 0.881 (R^2^ = 0.987). The slope (48 mV/pH) is close to the theoretical value (59 mV/pH), indicating an electrochemical process involving an equal number of electrons (e^−^) and protons (H^+^). 

#### 3.4.2. Effect of Scan Rates

**Figure 7:**
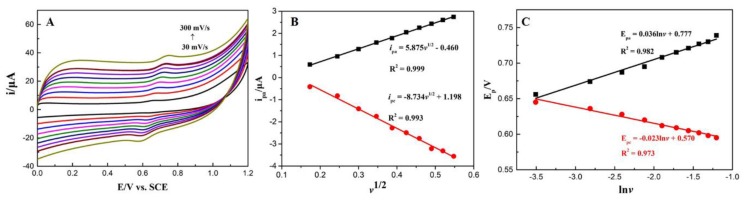
(**A**) Cyclic voltammograms of 10 μM of Allura Red at various scan rates. (**B**) Linear plots of anodic and cathodic peak currents of Allura Red against the square root of scan rates (*v*^1/2^); (**C**) Linear plots of anodic and cathodic peak potentials against the Napierian logarithm of scan rates (ln*v*).

**Scheme 1:**
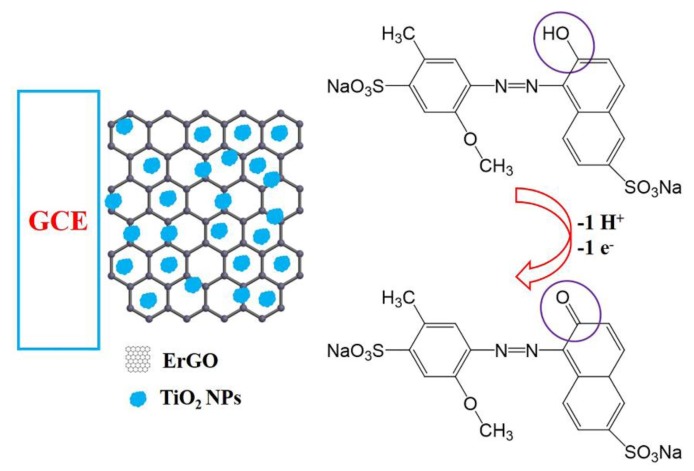
Schematic diagram of the electrochemical mechanism for the redox of Allura Red at TiO_2_/ErGO/GCE.

To understand the electrochemical mechanism for the redox of Allura Red, cyclic voltammograms of 10 μM Allura Red were measured by TiO_2_/ErGO/GCE at various scan rates. Cyclic voltammograms at different scan rates are shown in Figure 7A. A pair of redox peaks are observed at all scan rates with the ratio of i_pa_/i_pc_ approaching 1, demonstrating that a quasi-reversible process occurs at TiO_2_/ErGO/GCE. The anodic peak currents gradually increase with the increasing of scan rates. In addition, as the scan rates speed up, the anodic peaks of Allura Red shift positively, while the cathodic peaks move to the reverse direction. As shown in Figure 7B, there are good linear relationships between anodic/cathodic peak currents (i_pa_ or i_pc_) and the square root of scan rates (*v*^1/2^). Their linear equations are i_pa_ (μA) = 5.875*v*^1/2^ (V/s) − 0.460 (R^2^ = 0.999) and i_pa_ (μA) = 5.875*v*^1/2^ (V/s) − 0.460 (R^2^ = 0.993), respectively. This result indicates that the redox of Allura Red is a typical diffusion-limited process. Moreover, the anodic or cathodic peak potentials are highly correlated to the Napierian logarithm of scan rates (Figure 7C). Their linear regression equations can be expressed as E_pa_ (V) = 0.036ln*v* (V/s) + 0.777 and E_pc_ (V) = −0.023ln*v* (V/s) + 0.570, with a correlation coefficient (R^2^) of 0.982 and 0.973, respectively. As for a diffusion-limited quasi-reversible process, the relationship between redox potential (E_pa_ or E_pc_) and ln*v* should follow Laviron’s equations [58,59].

(1) $$E_{\mathrm{pa}}=E_{0}^{\prime }+\frac{RT}{(1-\alpha )nF}[0.78+\ln(D^{1/2}k_{s}{}^{-1})-\frac{RT}{2(1-\alpha )nF}]+\frac{RT}{2(1-\alpha )nF}\ln v$$

(2) $$E_{\mathrm{pc}}=E_{0}^{\prime }-\frac{RT}{\alpha nF}[0.78+\ln(D^{1/2}k_{s}{}^{-1})-\frac{RT}{2\alpha nF}]\ln v-\frac{RT}{2\alpha nF}\ln v$$

(3) $$\ln k_{s}=\alpha \ln(1-\alpha )+(1-\alpha )\ln\alpha -\ln\frac{RT}{nFv}-\frac{(1-\alpha )n\alpha \Delta E_{p}}{RT}$$

where α is the charge transfer coefficient, *n* is the electron transferred number, and other symbols have the usual meanings. Combining the linear relationships with Lavrion’s equations, the charge transfer coefficient (α) is calculated as 0.6. Then, the electron transfer number (*n*) is found to 0.89 ≈ 1.0, indicating a one-electron transferred electrochemical process of Allura Red at TiO_2_/ErGO/GCE. Based on the earlier discussion that the electrochemical oxidation of Allura Red at TiO_2_/ErGO/GCE was a process related to an equal numbers of electrons and protons, it’s deduced that the reaction mechanism of Allura Red involves one electron and one proton transfer, which is caused by the phenolic hydroxy (Scheme 1). The reaction mechanism is in good agreement with the previous report [4].

#### 3.4.3. Effect of Accumulation Parameters

**Figure 8:**
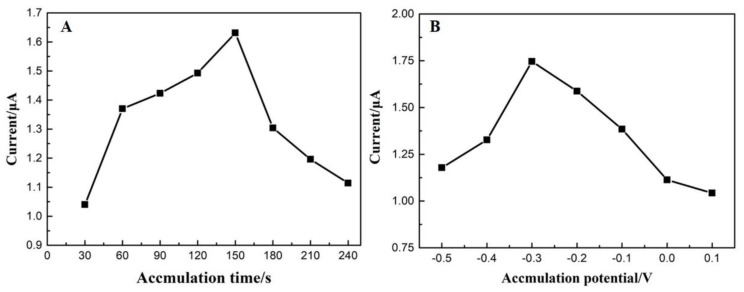
Influence of accumulation time (**A**) and accumulation potential (**B**) on the anodic peak currents of Allura Red.

The effect of accumulation time as well as potential were also investigated (Figure 7). As shown in Figure 8A, the anodic peak currents of Allura Red gradually increase for 150 s; then, they suddenly decrease with the further extension of accumulation time. So, 150 s was recommended as the optimal accumulation time. When the accumulation potentials were changed from −0.5 V to −0.3 V, their anodic peak currents gradually increase. However, they slowly decrease when the accumulation potential went beyond −0.3 V (Figure 8B). Hence, −0.3 V was chosen as the optimal accumulation potential. The optimal accumulation parameters (−0.3 V, 150 s) were adopted in the following measurements.

### 3.5. Selectivity, Reproducibility, and Stability

**Figure 9:**
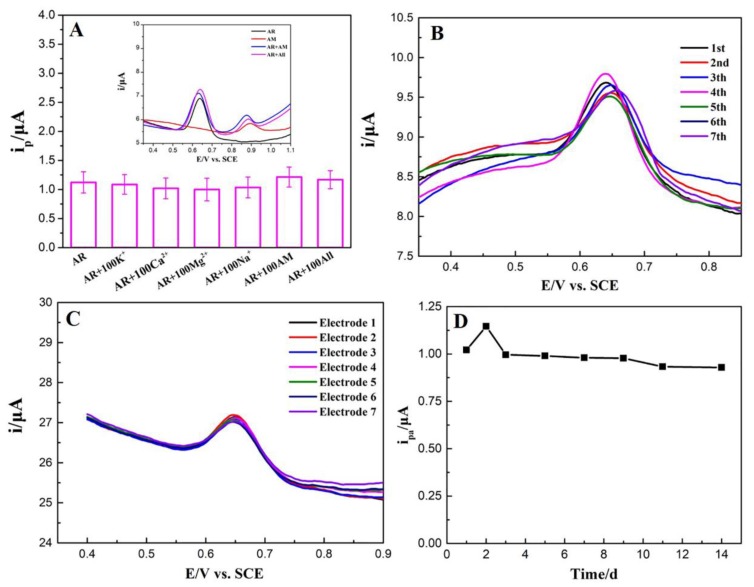
(**A**) Anodic peak current of Allura Red in the presence of 100-fold K^+^, Na^+^, Ca^2+^, Mg^2+^, and amaranth; (**B**) Seven successive measurements of Allura Red at the same TiO_2_/ErGO/GCE; (**C**) Anodic peak currents of Allura Red recorded at seven TiO_2_/ErGO/GCEs; (**D**) Anodic peak currents of Allura Red were regularly recorded during two weeks. The inset in Figure 9A is DPV of Allura Red in the existence of 100-fold amaranth and all interfering specifies. GCE: glass carbon electrodes.

To assess the selectivity of the proposed sensor, the DPV responses of Allura Red in the presence of common inorganic metal ions (e.g., K^+^, Na^+^, Ca^2+^, Mg^2+^) and amaranth were also measured. The relative errors lower than 5% in the coexistence of 100-fold of these potential interfering substances (Figure 9A), indicating excellent anti-jamming performance. It is worth pointing out that the anodic peaks of Allura Red and amaranth are well separated with rather wide peak-to-peak separation (∆E_p_) of 0.252 V. Moreover, the andic peak current of Allura Red is hardly changed even in the existence of 100-fold amaranth. The phenomenon also shows that it is feasible to detect Allura Red and amaranth simultaneously. To check repeatability, seven successive measurements were also carried out. The relative standard deviation (RSD) is 5.46%, suggesting that TiO_2_/ErGO/GCE have good repeatability (Figure 9B). Then, anodic peak currents of Allura Red were recorded at seven TiO_2_/ErGO/GCEs that were fabricated by the same procedure, aiming to evaluate electrode reproducibility (Figure 9C). The RSD of 4.83% is acceptable, demonstrating electrode fabrication that has good reproducibility. Finally, the anodic peak currents of Allura Red were monitored during two weeks. After two weeks, the anodic peak current of Allura Red retains 90.9% of its original value, demonstrating good stability (Figure 9D). 

### 3.6. Standard Curves, Detection Range, and Detection Limit

**Figure 10:**
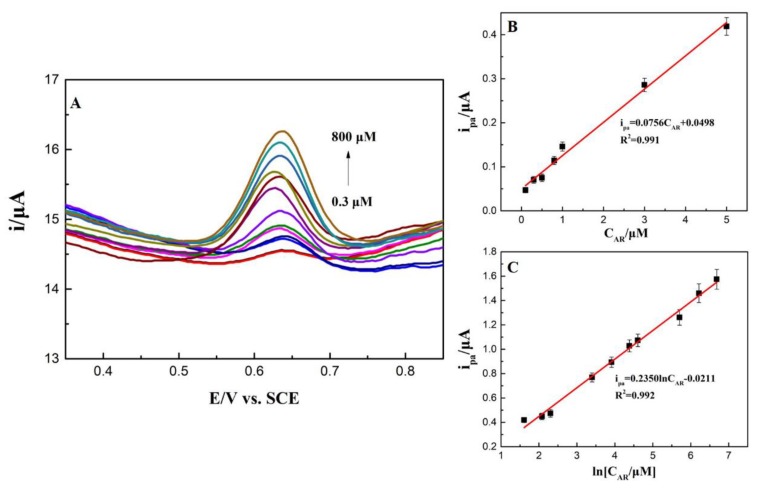
(**A**) DPV curves of Allura Red with various concentrations; (**B**) Linear plot of anodic peak currents of Allura Red versus their concentrations ranging from 0.3 to 5.0 μM; (**C**) Linear plot of anodic peak currents of Allura Red versus Napierian logarithm of their concentrations varying from 5.0 to 800 μM.

DPV curves of different concentrations of Allura Red are plotted in Figure 10A. Clearly, the response anodic peak currents of Allura Red increase with their concentrations. At a low concentration domain (0.3–5 μM), the anodic peak currents of Allura Red are linearly correlated to their concentrations (Figure 10B). The corresponding linear equation can be expressed as i_pa_ (μA) = 0.0756*C*_AR_ (μM) + 0.0498 (R^2^ = 0.991). However, the anodic peak currents of Allura Red are positively proportional to the Napierian logarithm of their concentrations (ln*C*_AR_) ranging from 5 to 800 μM (Figure 10C). The standard deviation (σ) for 10 parallel measurements in bank solution is 0.001276. Therefore, the detection limit (LOD) is estimated to 0.05 μM (LOD = 3σ/s). The analytical performances including detection range and detection limit are almost comparable to previous reported modified electrodes (Table 1). In addition, TiO_2_/ErGO nanohybrids possess the characteristics of low cost, available raw materials, and easy preparation, which is very advantageous for commercial applications.

### 3.7. Determination of Allura Red in Actual Samples 

In order to verify the workability, the level of Allura Red in milk drink samples was detected by our proposed TiO_2_/ErGO/GCE using DPV. Liziyuan drinking was diluted to 100-fold with 0.1 M PBS solution (pH = 7.0). The determination results are summarized in Table 2. The concentration of Allura Red in the Liziyuan milk drink sample is 14.28 μM (7088 μg/L). Obviously, the level of Allura Red in soft drink is far lower than the maximum permitted dosage (0.1 g/kg) specified in GB 2760-2014. To further verify the accuracy and precision of the proposed TiO_2_/ErGO/GCE, a series of known concentration solutions of Allura Red were spiked to the milk drink samples. The RSD is lower than 5.0% and recoveries range from 98.42% to 106.0%, confirming the feasibility for the Allura Red determination in real samples. In short, the proposed TiO_2_/ErGO/GCE shows tremendous application prospects on the determination of Allura Red in real samples such as beverages and food.

Based on the detected values, it is easy to estimate the amount of Liziyuan milk drink that an individual must drink to reach Allura Red’s ADI. For instance, if an individual weighs 60 kg, the amount of Liziyuan milk drink that must consume is 59 L per day. This means that the volume required to reach the ADI value is so large that the potential side effects on consumers health are negligible, even if it is consumed daily.

## 4. Conclusions

In this paper, a novel electrochemical sensor based on TiO_2_/ErGO nanocomposites were developed for the sensitive detection of Allura Red. TiO_2_/ErGO nanocomposites were synthesized by the hydrolysis of titanium sulfate in graphene oxide suspension and in situ electrochemical reduction. The electrochemical characterization of the proposed sensor was performed by cyclic voltammetry and electrochemical impedance spectroscopy with [Fe(CN)_6_]^3−/4−^ as the redox pair. TiO_2_/ErGO nanohybrids showed the largest electroactive surface area and lowest charge transferred resistance (R_ct_). As a result, TiO_2_/ErGO/GCE showed remarkable electrocatalytic activity toward Allura Red, which was mainly due to the synergistic effect between TiO_2_ NPs and ErGO nanosheets. The anodic peak currents of Allura Red were linearly correlated with their concentrations in the range of 0.3 to 5.0 μM. However, the linear relationship was changed to a semi-logarithmic relationship at a higher concentration region (5.0–800 μM). The detection limit (LOD) was estimated to 0.05 μM at SNR = 3. Finally, the Allura Red in soft milk drink was detected by the proposed TiO_2_/ErGO/GCE with satisfactory recovery. Featured with the fascinating merits including high sensitivity as well as good anti-jamming performance, repeatability, reproducibility, and stability, the proposed TiO_2_/ErGO/GCE nanocomposites have become an attractive candidate for the detection of food colorants.

## Figures and Tables

**Table 1:** Comparison of electroanalytical methods for the determination of Allura Red. LOD: detection limit.

Methods	Electrodes	Peak Potential (V)	Detection Range (μg/L)	LOD (μg/L)	Ref
DPV	MWCNT/GCE	0.68	50–600	24.8	[29]
SWV	CoO_x_/CPE	−0.25	49.6–496	24.8	[31]
DPV	MWCNT/GCE	0.72	496–4468	7.0	[28]
SWV	IL-EGPE	0.72	500–5000	0.89	[30]
DPV	SbF/SPCE	−0.68	500–2500	149	[27]
SWV	CoC/CPE	0.85	59.6–1489	39.7	[32]
SWV	GCE with CPB	0.91	29.8–5709	14.9	[57]
SWV	PAMI/GCE	−0.18	4964–148,920	695	[24]
DPV	TiO_2_/ErGO/GCE	0.62	149–2482; 2482–397,120	24.8	This work

**Table 2:** Determination results of Allura Red in milk drinking samples. RSD: relative standard deviation.

Samples	Detected (μM)	RSD (%)	Added (μM)	Found (μM)	RSD (%)	Recovery (%)
Milk drinking	14.28	4.26	1	15.34	2.81	106.0
	14.28	4.26	10	24.69	0.85	104.1
	14.28	4.26	100	112.7	4.86	98.42
